# Immune-Complex Mimics as a Molecular Platform for Adjuvant-Free Vaccine Delivery

**DOI:** 10.1371/journal.pone.0060855

**Published:** 2013-04-23

**Authors:** Ilaria Pepponi, Elena Stylianou, Craig van Dolleweerd, Gil Reynolds Diogo, Matthew J. Paul, Pascal M. W. Drake, Julian K.-C. Ma, Rajko Reljic

**Affiliations:** Infection and Immunity Research Centre, St. George's University of London, London, United Kingdom; Fundació Institut d’Investigació en Ciències de la Salut Germans Trias i Pujol. Universitat Autònoma de Barcelona. CIBERES, Spain

## Abstract

Protein-based vaccine development faces the difficult challenge of finding robust yet non-toxic adjuvants suitable for humans. Here, using a molecular engineering approach, we have developed a molecular platform for generating self-adjuvanting immunogens that do not depend on exogenous adjuvants for induction of immune responses. These are based on the concept of Immune Complex Mimics (ICM), structures that are formed between an oligomeric antigen and a monoclonal antibody (mAb) to that antigen. In this way, the roles of antigens and antibodies within the structure of immune complexes are reversed, so that a single monoclonal antibody, rather than polyclonal sera or expensive mAb cocktails can be used. We tested this approach in the context of *Mycobacterium tuberculosis* (MTB) infection by linking the highly immunogenic and potentially protective Ag85B with the oligomeric Acr (alpha crystallin, HspX) antigen. When combined with an anti-Acr monoclonal antibody, the fusion protein formed ICM which bound to C1q component of the complement system and were readily taken up by antigen-presenting cells *in vitro*. ICM induced a strong Th1/Th2 mixed type antibody response, which was comparable to cholera toxin adjuvanted antigen, but only moderate levels of T cell proliferation and IFN-γ secretion. Unfortunately, the systemic administration of ICM did not confer statistically significant protection against intranasal MTB challenge, although a small BCG-boosting effect was observed. We conclude that ICM are capable of inducing strong humoral responses to incorporated antigens and may be a suitable vaccination approach for pathogens other than MTB, where antibody-based immunity may play a more protective role.

## Introduction

Selection of safe and effective adjuvants is a major obstacle to protein-based vaccine development. For maximum protection against many viral and bacterial infections, neutralising antibodies and robust cellular effectors are likely to be needed at the portal of entry as well as systemically. However, development of effective subunit vaccines has been greatly impeded by the lack of appropriate adjuvants. A wide range of experimental adjuvants have been evaluated in animal models, including ADP-ribosylating bacterial enterotoxins (cholera toxin and heat labile enterotoxin) and mutant variants such as LTK63 and LTR72, synthetic CpG oligodeoxynucleotides, as well as delivery systems with adjuvanting properties including micro- and nano-particles and immune modulators. Adjuvant development thus remains one of the focal points in TB vaccine research field and a significant number of these molecules are currently undergoing human safety clinical trials (recently reviewed in [1]). However, the only adjuvants currently licensed for human use are aluminum compounds (e.g. Alum), MF59 and monophosphoryl lipid A (MPL), reflecting the inherent difficulties in adjuvant development and licensing. Hence, generating vaccines with built-in adjuvanticity that would not rely on exogenous adjuvants could significantly speed up the process of vaccine development and testing, including the clinical trials stage. Here, we explore the potential of immune complexes as the adjuvant-free vaccines.

It has long been known that primary and secondary antibody responses to model antigens can be enhanced by immunization with immune complexes (IC) [2]–[4]. Similar enhancement has also been demonstrated with viral antigens including Venezuelan equine encephalomyelitis virus vaccine [5], Hepatitis B surface antigen [6], HIV gp120 [7], and simian immunodeficiency virus (SIV) gp120 [8]. Importantly, it is now well established that IC can be cross-presented by antigen-presenting cells (APC) and can stimulate potent MHC class I as well as class II – restricted T cell responses [9], [10]. Indeed, antibody-mediated enhancement of SIV Gag antigen processing and cross presentation was recently demonstrated [11]. IC can enhance immune responsiveness through several mechanisms [12], including activation of the complement cascade [13], [14] and promoting of the Fc receptor-mediated recognition by APC [6], [15]. It has also been suggested that, through binding to Fc receptors and complement receptors, the IC localize on the surface of follicular dendritic cells (FDCs), which play an important role in selection and affinity maturation of B cells, or that the complexes might directly stimulate B cells via their complement receptors [16]. Antibody binding to antigen also leads to protection of the antigen from proteolysis intracellularly [17] and extracellularly [18], and this can lead to modulation of antigen processing, as well as antigen presentation [19]–[21]. These properties of IC raise the prospect of using them for vaccination, with the significant advantage that an additional adjuvant may not be required.

However, conventional preparation of IC is not applicable for vaccine development, as it relies on the use of either polyclonal antisera or expensive cocktails of monoclonal antibodies (mAbs) to achieve complexing with a given antigen. Thus, the complexity of formulation does not lend itself to pharmaceutical development. To solve this problem, Chargelegue and co-workers [22] described, for the first time, the production of recombinant IC in transgenic tobacco plants, by expressing tetanus toxin fragment C fused to a cognate mAb. The design of the recombinant IC fusion molecule resulted in the expression of antibody-antigen at the 1∶2 ratio (an antigen molecule was fused to each antibody heavy chain). Moreover, it was demonstrated in immunisation studies [22] that serum antibody responses leading to protective immunity were induced without additional adjuvant. In a similar fashion, Phoolcharoen *et al.* transiently expressed Ebola-based immune complexes in plants by genetically fusing Ebola GP1 glycoprotein subunit to a specific antibody heavy chain. Mice immunised with Ebola IC developed high titres of Ebola-specific IgG antibodies [23]. Thus, these studies show that IC might represent an attractive immunisation strategy if they could be produced in recombinant form, which is a prerequisite for a large scale good-manufacturing practice (GMP) production.

**Figure 1:**
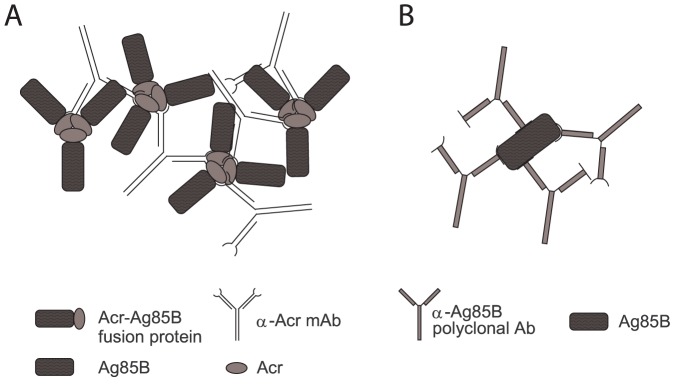
Schematic representation of immune complex mimics (ICM) based on Acr-Ag85B fusion protein and an anti-Acr mAb (A) and the classical immune complexes (IC) based on Ag85B antigen of MTB and polyclonal Abs (B). For ICM, the fusion protein is depicted as a trimer, which is one of the predominant molecular forms for Acr in solution. Each mAb molecule must bind to a different monomer unit of Acr (A); in contrast, polyclonal Abs can bind to the same Ag85B molecule (B).

Here, we describe a molecular engineering approach for generating recombinant IC mimics (ICM), by reversing the roles of the antigen and antibody within the structure of IC. Unlike the classical immune complexes which typically contain an antigen and several (i.e. a minimum of 2) polyclonal antibody molecules bound to different epitopes, the ICM consist of an oligomeric antigen and multiple copies of a single monoclonal antibody, as schematically depicted in Fig.1A. The resulting complex could be expected to be functionally indistinguishable from the true IC (Fig.1B) but is based on a single mAb rather than a cocktail of mAbs or polyclonal sera, making a standardised, large-scale preparation of these molecules feasible. The added advantage of the ICM approach is that it is possible to include additional antigens by genetically fusing them to the selected oligomeric protein. We generated such molecules based on two mycobacterial antigens and a single mAb, and tested the resulting ICM for immunogenic potential and protective capacity in the context of MTB infection.

## Materials and Methods

### Cloning of Acr, Ag85B and Acr-Ag85B MTB antigens

The Acr coding sequence (plasmid containing the ACR gene was kindly provided by Dr. Kris Huygen, Institute Pasteur, Brussels, Belgium) was amplified by PCR using ACR-For: 5'-CA GGA TCC CAT ATG GCC ACC ACC CTT CCC GTT CAG-3' and ACR-Rev: 5'-AA AGC GGA TCC TCA GTT GGT GGA CCG GAT CTG-3' primers which contain *Nde*I and *Bam*HI recognition sequences, respectively. After digestion with *Nde*I and *Bam*HI, the fragment was cloned into pET-15b plasmid (Stratagene). Similarly, Ag85B was generated using the primers Ag85B-For: 5′-CAG GAT CCC ATA TGT TCT CTC GTC CTG GTT TG-3′ and Ag85B-Rev: 5′-AAA GCG CCT CAA CCT GCT CCC AAA GAA GA-3′, with the pGEM-Ag85B-ESAT6 vector (kindly provided by Dr. Roger Frutos, Campus International de Baillarguet, France) as the template; the PCR product was then cloned into *Nde*I and *BamH*I linearised pET15b vector. The vector contains an IPTG (Isopropyl β-D-1-thiogalactopyranoside)-inducible T7 polymerase promoter and a generic His-tag sequence to assist purification of recombinant proteins. To generate the Acr-Ag85B fusion protein, the Acr gene was first amplified by PCR as before, except the reverse primer was 5'-AA AGC GGA TCC ACC GCC TGC GGC CGC GTT GGT GGA CCG GAT CTG AAT GTG CTT-3'. This primer contains the restriction sequence for *Bam*HI, to allow cloning of Ag85B, and lacks the stop codon to allow translation of the second TB antigen. The obtained PCR product was cloned into *Nde*I*/Bam*HI-cleaved pET-15b vector. To clone Ag85B in tandem with Acr, the obtained pET-15b:ACR was digested with *Bam*HI. The Ag85B-coding DNA fragment was amplified by PCR using the primers Ag85B-For (as above) and Ag85B-Rev: 5'-AA AGC GGA TCC TCA ACC TGC TCC CAA AGA AGA-3', each containing a *Bam*HI site. After cloning of the Ag85B sequence into the *Bam*HI-linearised and calf intestinal phosphatase (CIP)-treated pET-15b:ACR plasmid, orientation of the inserted gene was verified by restriction enzyme analysis and sequencing; the plasmid was then used to transform BL21 *E. coli* cells for expression.

### Expression and purification of recombinant proteins

A 1/50 volume of an overnight culture was added to LB broth supplemented with 50 µg/mL of carbenicillin and 34 µg/mL of chloramphenicol. Growth was monitored by measuring optical density at 600 nm. IPTG was added to a final concentration of 0.5 mM when OD_600_ reached 0.6. The cell culture was then incubated for 4 h before harvesting by centrifugation (5000 g) for 15 minutes at 4°C. Cell pellet was re-suspended in 24 mL of phosphate-buffered saline (PBS) pH7.2, containing 0.05 mg/mL PMSF(phenylmethylsulfonyl fluoride), a protease inhibitor, and 1 mg/mL of lysozyme, and incubated on ice for 30 minutes. In order to completely disrupt cells, Triton X-100 was added to 1% (v/v), together with DNase (5 µg/mL), and the solution was incubated at room temperature for 20–30 minutes. At this stage the whole cell extract (WCE) was used for the evaluation of protein expression, or processed for further purification as follows. The WCE was centrifuged at 20000 g for 30 minutes at 4°C; supernatant (soluble WCE, sWCE), was removed and the pellet (insoluble WCE, iWCE), was dissolved in 8 M urea to solubilise proteins contained within the inclusion bodies. The iWCE obtained from 1 L culture was dissolved in 40 mL of DIMAC-5 (8 M urea, 5 mM imidazole, 0.5 M NaCl, 20 mM Tris-HCL, pH 7.9) and filtered through a 0.22 µm filter. The filtered sample was applied to chelating sepharose Ni-chromatography column (Pharmacia) equilibrated in DIMAC-5. To eliminate contaminants and impurities the column was washed with 40 column volumes (cv) of DIMAC-5 and 20 cv of DIMAC-20 (same as DIMAC-5 except 20 mM imidazole), followed by a 20 cv of 50% v/v 1,2-hexandiol, to remove LPS. Bound protein was eluted in DIMAC-100 (with 100 mM imidazole), concentrated and dialysed against 10 mM ammonium carbonate buffer, pH 7.7, containing 0.1 mM CaCl_2_.

### SDS-PAGE and Western blotting

Protein samples were mixed with 1/4 volume of 4xSDS-loading buffer, boiled for 3 minutes and loaded onto a 4–12% gradient polyacrylamide gel (Tris-glycine; BioRad). Following separation of proteins, gels were either stained with Coomassie Blue or subjected to Western blot analysis. This was performed by transferring proteins to a nitrocellulose membrane using a semi-dry transfer system (Hoefer™ TE70, Amersham Biosciences), followed by blocking of blots in 5% (w/v) non-fat dried milk in PBS at 4°C and incubated at room temperature for 2 h with appropriate primary and secondary antibodies diluted in PBS-dried milk 5% (w/v). The primary antibody for Acr was TBG65 mAb and the secondary antibody was a goat anti-mouse IgG1 horseradish peroxidase-conjugated antibody (1∶1000; Jackson Immuno Research); for Ag85B, a rabbit polyclonal anti-Ag85B antibody (1∶1000) was used, followed by a donkey anti-rabbit monoclonal antibody conjugated to horseradish peroxidase (1∶1000; Jackson Immuno Research). In some instances, the fusion protein was detected by a mouse monoclonal anti-polyHistidine antibody (SIGMA) conjugated to horseradish peroxidase (1∶1000). After washing of blots with PBS, they were developed using enzymatic chemiluminescence (ECL-Enhanced Chemiluminescence, Amersham Biosciences).

### Cross-linking of polymeric proteins

50 µg of protein in PBS (Acr, Ag85B-Acr or Ag85B as the negative control, all at 1 mg/ml) was crosslinked by addition of 5 mM Disuccinimidyl suberate (DSS, Pierce). Following addition of the crosslinker, the samples were incubated for 30 minutes at room temperature, before the reaction was quenched by addition of SDS-PAGE loading buffer and 100 mM DTT (dithiothreitol). Crosslinked protein was then analysed by SDS-PAGE and Western blotting.

### TBG65 mAb purification

Mouse TBG65 hybridoma cell line (generated by Prof. Juraj Ivanyi, King's College London, [24]) was grown in RPMI complete medium (Invitrogen). A CELLine CL-1000 (Integra Biosciences) flask, specifically designed for high-yield antibody production, was inoculated with 2.5×10^7^ cells undergoing exponential growth. Cells were incubated in a tissue culture incubator (37°C, 5% CO_2_, 90% relative humidity) and partially harvested every 3–4 days over a period of 1 month. Antibody purification was performed by affinity chromatography on an Affigel-15 (Bio-Rad) column with immobilised Acr. After washing the column with 30 cv of PBS, antibody was eluted in 0.1 M glycine pH 2.5 and the fractions neutralised by addition of 1/10 volumes of 1 M Tris (uncorrected pH). Finally, protein was concentrated to 5 mg/ml and dialysed against PBS.

### Preparation of ICMs

The purified TBG65mAb and the Acr-Ag85B fusion protein were combined in PBS pH 7.2 at a molar ratio ranging from 1∶1 to 1∶20. The total protein concentration in the solution was kept below 1 mg/ml, to avoid formation of large aggregates that could precipitate. After 1 h incubation at room temperature the ICMs were placed on ice and subsequently kept at 4°C, prior to application. ICMs were tested for their functionality by a C1q binding ELISA and in a cell-binding assay.

### Complement C1q ELISA

96 well plates (Nunc Maxisorp Immuno™ plates) were incubated with 10 µg/mL of C1q (Calbiochem) component of the complement system, at 37°C for 2 h. Plates were blocked with PBS-2.5% BSA (4°C overnight) and then serial dilutions of ICM were added in a final volume of 50 µL/well and incubated at 37°C for 2 h. The plates were washed five times in distilled water, and the secondary antibody (goat anti-mouse IgG HRP-conjugated antiserum; The Binding Site) was added in PBS-2.5% BSA and again incubated at 37°C for 2 h. Detection was performed using TMB (3,3', 5,5"-tetramethylbenzidine) substrate (Sigma) and the absorbance was measured at 450 nm.

### Binding of ICMs to antigen-presenting cells

1×10^6^ spleen-derived APC, prepared as described by Pal *et al* [25], were re-suspended in 100 µL of PBS containing 5% BSA. ICMs (10 µg/mL) were added and cells incubated on ice for 2 h. Unbound protein was removed by repeated (3x) addition of the binding buffer and recovery of cells by centrifugation. Secondary anti-mouse IgG-FITC antibody (Sigma) was added at a concentration of 1 µg/mL and cells incubated for a further 1 h on ice. Cells were then washed 3 times in binding buffer as before, re-suspended in 1 mL of binding buffer and analysed for green fluorescence in a Becton-Dickinson flow cytometer. Ten thousand cells were counted and results expressed as percentage of cells positive for FITC staining.

### Immunisation of mice

Six to eight weeks old female Balb/c mice (Harlan, UK) were used in all immunisation experiments. The mice were kept under defined environmental conditions (12∶12 h light:dark cycle, 19–21°C, 55% relative humidity, pathogen free). Mice were immunised subcutaneously (s.c) with 50 µg of protein administered at the base of the tail, followed by a boost immunisation three weeks later. All administrations were standardised for the amount of Ag85B in the vaccine formulation. The 1∶20 antibody:antigen molar ratio in ICMs was used in all experiments, following initial testing of various ratios in a pilot experiment. In some experiments, mice were first immunised with 5×10^5^ BCG (s.c) followed by two s.c boosts with 50 µg ICMs at weeks six and eight. In all experiments, PBS or BCG (Pasteur strain, grown in 7H9 Middlebrook medium) immunised mice were used for comparison and mice immunised with Ag85B on its own (negative control) or together with cholera toxin (CT fragment b) adjuvant (positive control) were used to determine immune responses induced by ICMs.

### Humoral response

Specific humoral responses in sera of mice immunised with ICMs were tested by ELISA. At various time points during immunisation, blood was collected from the tail vein or by cardiac puncture (end of protocol) and fractionated to obtain sera. 96 well plates (Nunc Maxisorp Immuno™ plates) were coated with 10 µg/mL antigen solution (either Acr or Ag85B) in PBS for 2 h at 37°C. The plates were then washed with distilled water and blocked overnight with a solution of 2.5% BSA in PBS at 4°C. Serial dilutions of sera were added and plates incubated for 2 h at 37°C. After washing, peroxidase-conjugated detection antibodies (sheep anti-mouse, The Binding Site) diluted 1∶1000 in PBS-2.5% BSA were added, and incubated as before. Total antigen-specific IgG or IgG1and IgG2a subtypes were analysed. Following a further washing step, the binding was detected by adding 100 µL of TMB substrate (Sigma) to each well and the reaction was stopped with 50 µL/well of 2 M sulfuric acid. The absorbance at 450 nm was read on an ELISA plate reader (Sunrise Tecan).

### T cell proliferation and IFN-γ detection

Spleens were aseptically extracted from mice and kept on ice. The spleens were cut in small pieces and squeezed through a 70 µm cell strainer (BD Falcon™) in complete RPMI medium (Sigma) supplemented with 10% fetal bovine serum (FBS, Sigma), 100 U/mL penicillin and 0.1 mg/mL streptomycin (Sigma). The released cells were spun for 5 minutes at 190 g in a ROTINA 48R centrifuge (Hettich Zentrifugen) and pelleted. Supernatant was discarded and cells were re-suspended in 3 mL of red blood cell lysing buffer (Sigma) and incubated for 2 minutes at room temperature to eliminate the red blood cells. Complete RPMI medium was added to the cell suspension and the cells were spun again as above, followed by two additional washing steps. Cells were then counted and seeded into 96 well flat bottom plates (BD Falcon™) at a density of 3×10^5^ cells/well, in 200 µL medium. Three different stimuli were used: 5 µg/mL of concanavalin A (Sigma), 10 µg/mL of Ag85B and 10 µg/mL of Acr. Cells were incubated at 37°C for 48 h and a sample of medium taken for IFN-γ detection, using mouse IFN-γ kit, R&D systems, USA. The cultures were then pulsed with 1 µCi/well of ^3^[H]-thymidine (GE Healthcare) and incubated for a further 24 h before harvest. At harvest, cells were transferred onto a glass fiber filter (Wallac) using a TOMTEC harvester and fixed with a scintillator sheet (MeltiLex™ A, Perkin Elmer), prior to determining counts per minute (CPM) in a liquid scintillator counter (Wallac).

### Challenge of mice with H37Rv MTB

All pathogenic work was conducted in a containment level 3 laboratory in a Class 1 microbiological safety cabinet. Two weeks after the last immunisation (week 5 for homologous immunisation, week 10 for BCG-prime, ICM-boost), mice were lightly anaesthetized and challenged intranasally with 70,000 CFU (colony-forming units) of MTB laboratory strain H37Rv (kindly provided by P. Butcher at St George's, and grown in 7H9 Middlebrook medium). Four weeks later, mice were culled and lungs recovered. Organs were homogenised in 5 mL of distilled water using a Stomacher-80 biomaster (Seward) and serial dilutions plated out on Middlebrook 7H11 mycobacteriological plates. CFUs were counted three weeks later and the results expressed as logarithmic values using GraphPad software.

### Statistical analysis

Results were analysed by ANOVA followed by Tukey's (using GraphPad PRISM software) or in some instances by Student's t-test, for a single group-to-group comparison (i.e. BCG *vs* BCG+ICM). In all instances, p<0.05 was considered a statistically significant difference.

### Ethics Statement

Animal work was conducted at the St George's University of London (SGUL) Biological Research Facility which is a designated establishment for animal research. The work in this study was approved by the SGUL Ethical Research Committee, as part of the process of obtaining the UK Home Office animal project licence. Due care was taken at all times to minimise suffering of animals during the experimentation. Experimental end-points always preceded the onset of any visible symptoms of TB disease.

## Results

### Expression and molecular analysis of Acr, Ag85B and Acr-Ag85B

**Figure 2:**
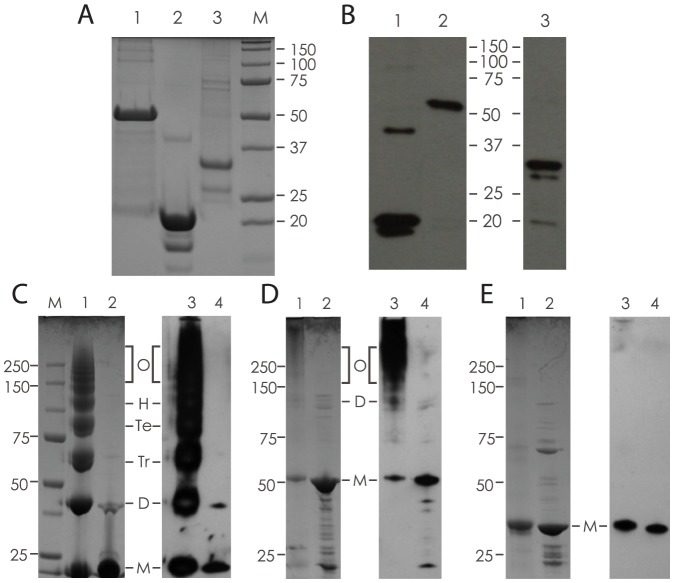
Expression, purification and chemical crosslinking of recombinant proteins. A) Coomassie Blue staining of purified, His-tagged proteins separated by 12% SDS-PAGE; 1. Acr-Ag85B (50 kDa), 2. Acr (20 kDa) and 3. Ag85B (32 kDa). B) Western blot analysis using antigen-specific antibodies; 1. Acr, 2. Acr-Ag85B and 3, Ag85B (1 and 2 probed with anti-Acr mAb TBG65; 3 probed with rabbit anti-Ag85B serum). C) Chemical crosslinking of Acr; shown is a Coomassie-stained (1, and 2) or Western blot (3 and 4) analysed sample with crosslinker (1 and 3) or without crosslinker (2 and 4). Letters indicate various molecular forms based on expected size (M-monomer, D-dimer, Tr-trimer, Te-tetramer, H-hexamer, O-oligomers). D) Chemical crosslinking of Acr-Ag85B fusion protein; shown is a sample with (1 and 3) or without (2 and 4) crosslinker. Letters indicate various molecular forms as for Acr (C). E) Chemical crosslinking of Ag85B (internal control); Coomassie and Western blot analysis of a sample with (1 and 3) or without (2 and 4) crosslinker.

The recombinant proteins were expressed and purified from BL21 inclusion bodies, and analysed by SDS-PAGE and Coomassie staining (Fig.2A). Acr-Ag85B (lane 1) migrated as a 50 kDa protein band corresponding to the expected size of the fusion protein. Acr preparation (lane 2) contained a dominant 20 kDa protein band (slightly larger than expected size of 18 kDa, presumably due to presence of the His-tag) and two minor contaminants, one of which (the 40 kDa band) corresponds to Acr dimer. Ag85B (lane 3) appeared as a dominant 32 kDa protein band, with some lower molecular weight contaminants also present, but not recognised by anti-Ag85B antibodies (see further below). To confirm the identity of the purified proteins, Western blot analysis was performed, using anti-Acr and anti-Ag85B antibodies (Fig.2B). The results show that all three dominant protein bands were recognised with corresponding specific antibodies, and that the Acr preparation (lane 1) also contained an additional immune-reactive 40–42 kDa protein band, possibly non-dissociated dimer.

To verify the oligomeric status of Acr and Acr-Ag85B, chemical crosslinking using a short-length crosslinker (favouring intra- rather than inter-molecular linking) was performed and the proteins analysed by SDS-PAGE, followed by Coomassie staining or Western blotting. As can be seen in Fig.2C, Acr readily formed dimers, trimers, tetramers, hexamers and higher oligomers. The fusion protein of Acr and Ag85B also formed higher molecular forms, ranging in size from 100 kDa (dimer) to above 250 kDa, which were not well separated on the top of the 4–12% gel (Fig.2D). As expected, Ag85B on its own failed to form higher molecular forms in the presence of the crosslinker, as demonstrated by the lack of definable molecular bands, despite the monomeric band appearing slightly diminished on Coomassie staining (Fig.2E).

### Preparation and functional properties of ICM

**Figure 3:**
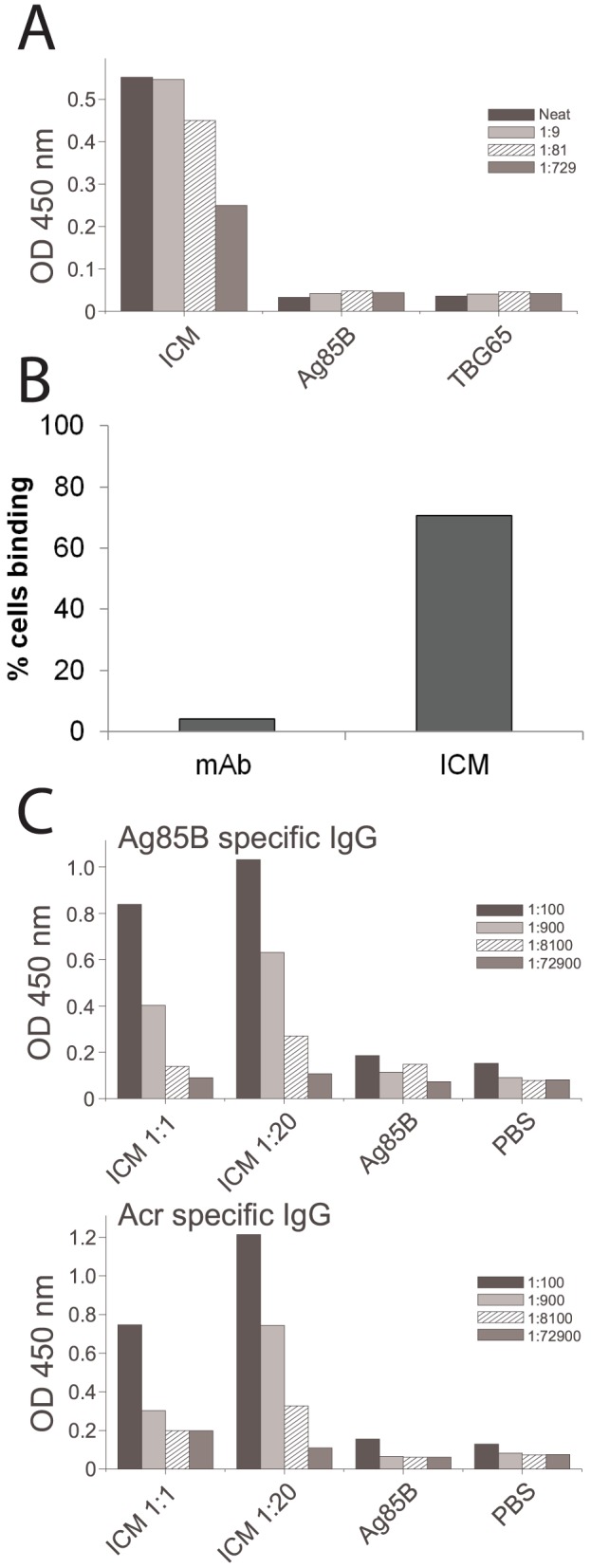
Functional evaluation of ICM in vitro and in vivo. A) Complement C1q binding ELISA; ICM were used at the 1∶20 antibody-antigen ratio and the neat sample contained 5 µg/m total protein (ICM) or the equivalent amount for individual components; each bar represents mean value from triplicate assays and the patterns indicate serial dilutions. B) Analysis of binding of ICM to spleen-derived APCs by flow cytometry; shown are the proportions of cells (out of 10,000 counted) that bound either mAb alone or ICM. C) Serum anti-Ag85B IgG responses from mice immunised with an equimolar (1∶1) or a low (1∶20) antibody-antigen ratio, twice at the base of the tail, at 3-week intervals. Mice were culled 3 weeks after the final immunisation. Shown are the mean values and corresponding serial dilutions from a pilot experiment (n = 3 mice).

ICM were prepared by combining the anti-Acr IgG1 mAb TBG65 and the Acr-Ag85B fusion protein at two different molar ratios (i.e. 1∶1 and 1∶20) in PBS buffer. To test the functional properties of ICM, we conducted two assays, namely complement C1q binding by ELISA and binding to the surface of antigen-presenting cells (APC) by flow cytometry. In the first assay, the ICMs were shown to bind the complement C1q component in a dose dependent manner while neither component alone showed any binding (Fig. 3A). For testing the ability of ICM to bind to APC, we generated mouse spleen derived APC as described previously [25]. Their phenotype was analysed by surface molecule staining and they were found to be positive for CD16/32 (93%), CD11b (98%), CD11c (65%) and MHC II (51%) (not shown), consistent with myeloid dendritic cells and macrophages. Following incubation with ICM, 71% of the APC were stained positive (Fig3B), while the cells incubated with the antibody alone showed only background staining.

### ICM-induced IgG responses in mice

**Figure 4:**
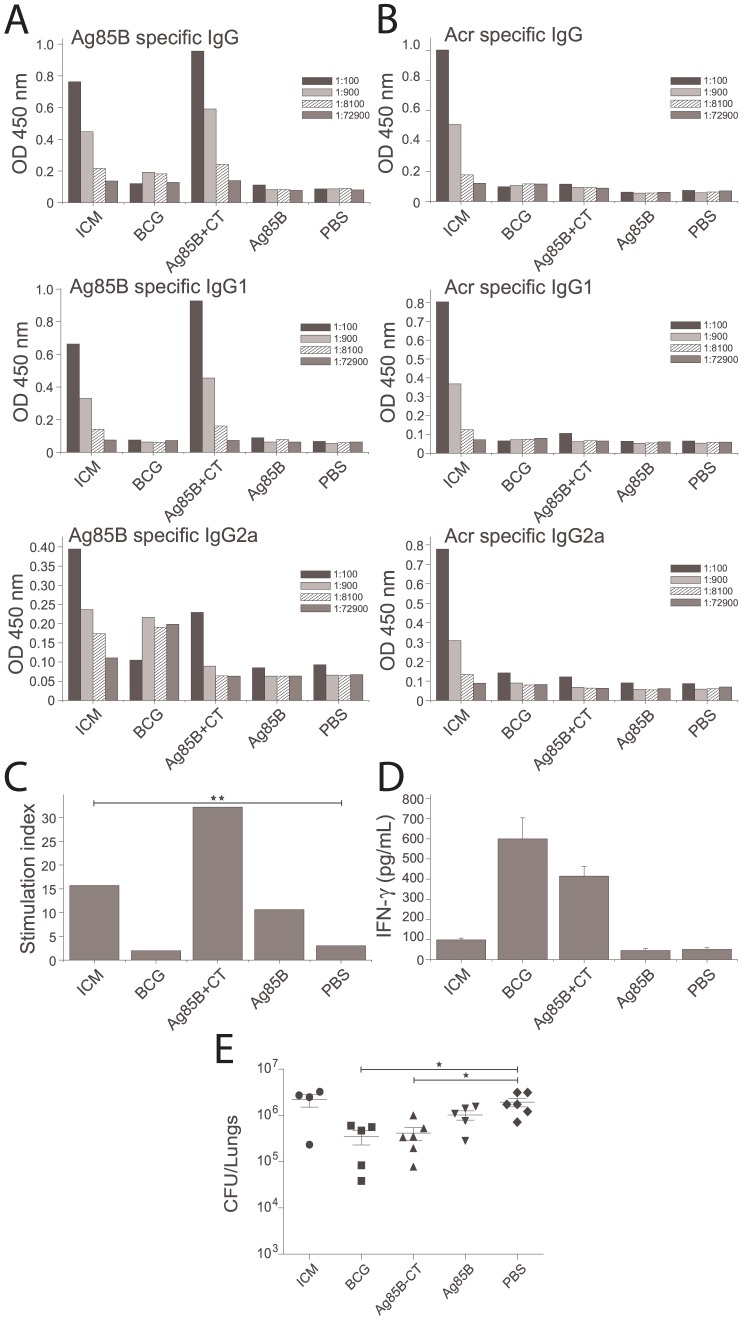
Immune responses and MTB bacterial counts in mice immunised with ICM. Mice were immunised with 50 µg ICM (1∶20 antibody antigen ratio) or with Ag85B alone (30 µg), Ag85B+CT, BCG and PBS; two weeks after the final immunisation mice were either culled and their tissues (blood and spleens) used for immunological evaluation, or challenged i.n. with 70,000 MTB H37Rv. * Indicates statistically significant difference (p<0.05). A,B) Ag85B (A) and Acr (B) specific IgG, IgG1 and IgG2a serum responses determined by ELISA; shown are the mean values from 3 mice analysed in triplicates and in serial dilutions (indicated by differing patterns). C) Splenocyte proliferation after *in vitro* stimulation with Ag85B, measured by ^3^[H]-thymidine incorporation and expressed as stimulation indices (specific/nonspecific proliferation); n = 3 animals. D) IFN-γ release in splenocyte cultures (as in C) measured by an IFN-γ ELISA based kit. E) Lung bacterial counts in immunised mice; shown are the counts for individual mice (n = 6, except in some groups due to death of animals before the end of the experiment) and the means +/− SEM for each group.

Following immunisation of mice, we analysed the IgG immune responses to both Ag85B and Acr in sera by antigen-specific ELISA. Initially, we tested different ratios of antibody/antigen within the ICM (i.e. 1∶1 or 1∶20) and found that the lower ratios induced a stronger humoral response (Fig.3C); hence the 1∶20 ratio was used in all subsequent experiments. Mice immunised with PBS, BCG or Ag85B alone showed no significant IgG response to either Ag85B or Acr (Fig.4A,B; final antibody titres shown only). In contrast, mice immunised with ICM, showed an Ag85B-specific IgG response comparable to that induced by formulating Ag85B with cholera toxin (CT). The end-point titre increased from 1∶100 (post-prime), to 8100 (after first boost), to 72900 (after second boost). A similar pattern was also observed for IgG responses to Acr. When analysing IgG subtype responses, both IgG1 and IgG2a were found to be present and with similar end-point titres, indicating a mixed type Th1/Th2 antibody response. When compared to the CT-adjuvanted group, ICM induced somewhat weaker IgG1 but higher IgG2a responses, consistent with the CT being a strong Th2 response promoting adjuvant.

### T cell response and IFN-γ production

At the end of the immunisation protocol, mice were sacrificed and the spleen cells recovered. Cell cultures were stimulated with Ag85B, medium alone or with 5 µg/ml Concanavalin A (not shown), as an internal control for proliferation. After 48 h, cell culture supernatant was analysed for IFN-γ, while the remaining culture was pulsed with [^3^H]-thymidine and analysed for radioactive incorporation 24 h later. Cell proliferation was expressed as stimulation index, obtained by dividing antigen-specific with medium-induced thymidine incorporation. As shown in Fig.4C, PBS and BCG immunised groups showed no splenocyte proliferation. Cells from animals immunised with Ag85B alone showed some antigen-specific proliferation and ICM induced a further proliferative response, although this was not statistically significantly different from Ag85B alone. However, the ICM-induced proliferation was statistically significantly higher than in the PBS group (p = 0.0037, Student's t test). On the other hand, cells from Ag85B+CT immunised animals showed twice the level of cell proliferation induced by ICM. A similar trend was observed for the IFN-γ response in culture supernatants (Fig.4D), except that the BCG-immunised animals showed the highest level of IFN-γ production.

### Bacterial load in MTB-infected mice

Four weeks after pathogenic challenge with MTB H37Rv strain (9 weeks from the start of immunisation), mice were culled and the bacterial load in their lungs determined by the standard CFU assay. As shown in Fig.4E, the groups were significantly different (p = 0.0018, one way ANOVA) but the only groups that showed a statistically significant reduction in bacterial load when compared to PBS-immunised mice, were BCG and Ag85B+CT (p<0.05, Tukey's test). ICM-immunised animals showed a similar bacterial number to the negative control.

### BCG-boosting potential of ICM

**Figure 5:**
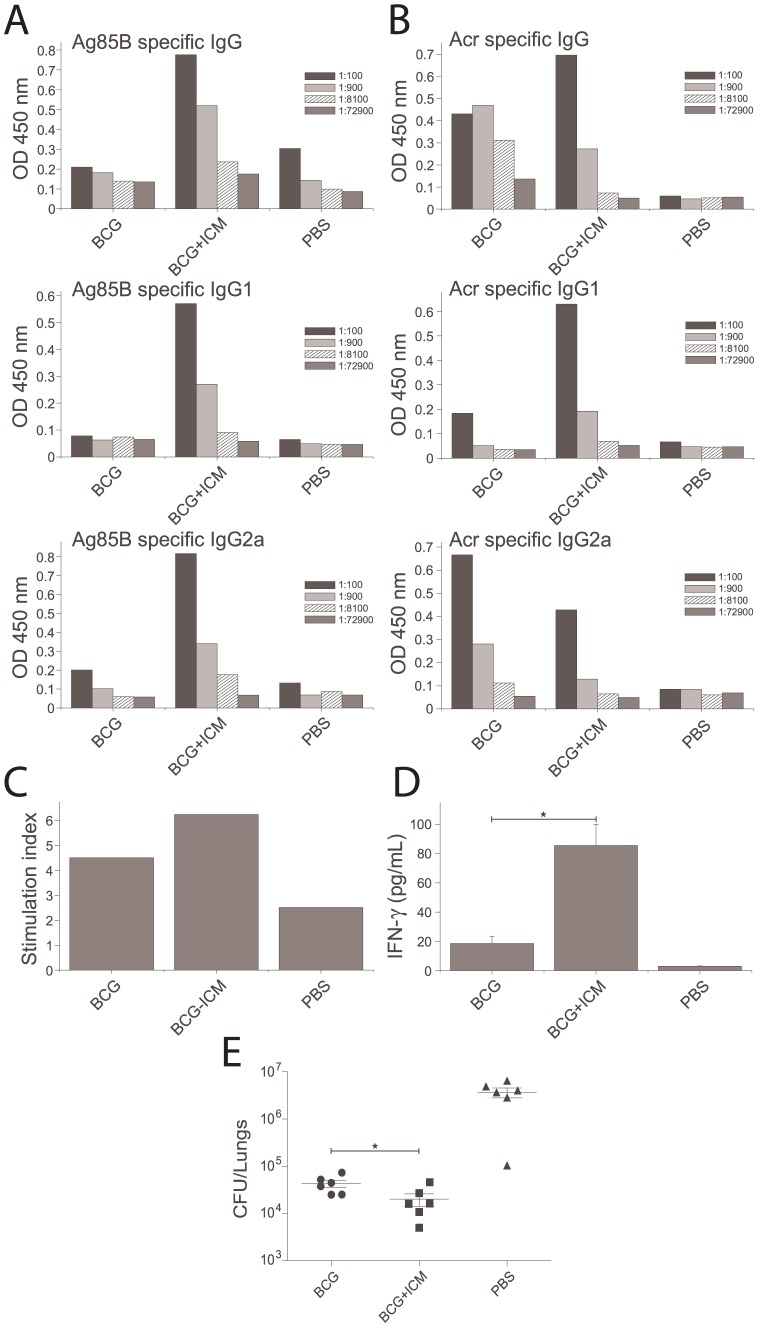
Immune responses and MTB bacterial counts in mice immunised with BCG and boosted with ICM. Mice were immunised s.c. with BCG and twice boosted with ICM six and eight weeks later; 2 weeks after the final boost, mice were either culled and their tissues (blood and spleens) used for immunological evaluation, or challenged i.n. with 70,000 H37Rv. * Indicates statistically significant difference (p<0.05). A,B) Ag85B (A) and Acr (B) specific IgG, IgG1 and IgG2a serum responses determined by ELISA; shown are the mean values from 3 mice analysed in triplicates and in serial dilutions (indicated by differing patterns). C) Splenocyte proliferation after *in vitro* stimulation with Ag85B, measured by ^3^[H]-thymidine incorporation and expressed as stimulation indices (specific/nonspecific proliferation); n = 3 animals. D) IFN-γ release in splenocyte cultures (as in C) measured by an IFN-γ ELISA based kit. E) Lung bacterial counts in immunised mice; shown are the counts for individual mice (n = 6) and the means +/− SEM for each group.

Since ICM failed to confer protection against MTB in immunised mice, they were subsequently tested as a boost vaccine to BCG. Mice were immunised with BCG (week 1) and boosted twice with ICM (weeks 6 and 8). Their humoral and cellular immune responses were analysed as before (week 10) and also the bacterial load in their lungs compared to BCG alone or the negative control (week 14). As shown in Fig.5, the ICM-boosted, BCG-primed group showed an enhanced IgG antibody response to Ag85B (5A) and Acr (5B), and a small though not statistically significant increase in T-cell proliferation (5C). A statistically significant (p<0.05) increase in IFN-γ production in response to Ag85B was measured (5D), although the proliferative response and cytokine secretion in all cultures was affected by reduced cell viability in this particular experiment. The bacterial load in the ICM-boosted animals, when compared to BCG-alone, was significantly lower (p = 0.037, Student's t test), but the magnitude of the effect (a 5-fold reduction) was relatively small (Fig.5E, representative of 2 similar experiments). Note also that the increased protection by BCG in this, compared to the experiment in Fig.4 may have been due to extension of immunisation time from 10 to 14 weeks.

## Discussion

This is a proof-of-principle study in which we investigated the immunogenic potential of recombinant immune complex mimics (ICM) as a novel molecular platform for adjuvant-free vaccine delivery. IC have many immunogenic properties that could be utilised in vaccine design. Unfortunately, the key obstacle for translating the potential of IC into a vaccine modality is that they cannot be produced reproducibly on a large scale and according to good manufacturing practices. Here, we describe a potential solution to that problem by reversing the roles of antibodies and the antigen within the structure of IC, without affecting their functionality. Importantly, such mimics of IC can be made reproducibly and on a large scale, as they are based on recombinant proteins and monoclonal antibodies. In this study, we generated ICM that incorporate a monoclonal antibody and two mycobacterial antigens, Ag85B and Acr, of which the latter is oligomeric and the target for the antibody moiety (mAb TBG65). TB is a prime example of a disease where development of a new vaccine has been very much impeded by the lack of suitable adjuvants, despite several antigens being shown to be potentially protective.

Ag85B was selected as previous evidence suggests that it has a protective potential [26] and is included in several vaccine candidates in clinical trials. Ag85B is strongly recognized by mouse Th1 cells [27], and frequently by human CD4^+^ T cells [28], during infection with MTB. Acr is a latent antigen of MTB expressed under hypoxic conditions and during the stationary growth phase [29]. It is also associated with stress response adaptation to growth in macrophages [30], [31] or in the presence of low nitric oxide concentration [32]. Acr is localised in the cell membrane [33] and it is a member of the heat shock protein family that exerts its chaperone activity by forming high molecular weight aggregates [29], [34]. Thus, Acr has been demonstrated to form polymeric structures in solution, via association of monomers through the N-termini [35]–[37]. Although this polymerising property of Acr was the key feature of this molecule that was exploited for generating ICM, this antigen might also contribute to protective immunity in TB. Acr is a dominant antigen preferentially recognised by latently infected individuals [38] and chronically infected mice developed a much stronger immune response specific for the DosR regulon antigens (which includes Acr) than acutely infected animals [39]. Both B and T cell epitopes have been identified [40]–[43] and anti-Acr antibodies were also shown to be effective in passive protection studies [44]–[46].

ICM were made by combining Acr-Ag85B fusion protein and a monoclonal mAb against Acr. They were then evaluated *in vitro* and in mice, for their immunogenic potential and protective capacity in the context of MTB infection. In the first *in vitro* assay, a complement C1q ELISA was performed to test whether ICM were able to activate the complement cascade. Only complexes which contain two or more antibody molecules can bind to C1q and activate complement via the classical pathway [47]. The result showed that ICM efficiently bound to C1q while neither component alone did. In the second *in vitro* assay, ICM were evaluated for their binding to APCs by flow cytometry analysis. Efficient binding to spleen-derived APCs was detected for ICM (ICM bound to 71% of cells), while TBG65 mAb on its own did not bind. These results demonstrate the potential of ICM as a new antigen delivery system for direct targeting of APCs, and enhanced antigen presentation to B and T cells. Indeed, it is now well established that dendritic cells can present exogenous antigens to not only helper, but also cytotoxic T cells, a phenomenon known as “cross-presentation” [48], [49]. Therefore, ICM carry the potential to induce a broad immune response to selected antigens in the absence of adjuvants [50].

It has been demonstrated that both the affinity of the antibody for the antigen and the antibody:antigen ratio are crucial for the generation of immunogenic IC [51]. TBG65 antibody used in ICM has a relatively high affinity for Acr (Kd = 9×10^8^) which should facilitate rapid formation of immune complexes. Indeed, we noted that at higher than equimolar ratios of antibody and antigen (i.e. 1∶1, 2∶1 etc.), the formed complexes grew too large and precipitated (not shown). The highest IgG response against Ag85B was obtained with the 1∶20 antibody:antigen molar ratio, which was statistically significantly higher than for the equimolar ratio. This was surprising as it was expected that an equimolar ratio would induce the strongest immune response, and other studies were based on an antibody:antigen ratio of 1∶2 (in the case of plant expressed IC; [22], [23] or higher (where mAb cocktail or polyclonal sera were used [9], [52]. Therefore, it is possible that the size of the ICM could be a limiting factor for the uptake by APCs. In this study, we generated ICM an hour prior to application and did not specifically test whether the size or immunogenicity of ICM would be altered after long-term incubation or storage. This is likely to depend on the oligomeric status of the antigen and the affinity of the mAb, and could be tested experimentally for each antigen-antibody combination.

ICM based on Acr-Ag85B fusion protein induced a strong humoral response to both antigens, which in the case of Ag85B was comparable to cholera toxin adjuvanted protein. Antibody responses were further analysed by measuring IgG1 and IgG2a isotype end-titres (associated with Th2- and Th1-type immune response, respectively), and both were found to be present at similar levels, indicating a mixed type response. T cell proliferative response and IFN-γ production after in vitro re-stimulation of splenocytes from ICM-immunised mice were higher than for antigen-alone or BCG-immunised groups; however, the relative levels were only moderate and this is the likely reason for the apparent failure of ICM to confer protection against pathogenic challenge on their own. However, some reduction of the lung bacterial load was observed when ICM were used to boost BCG, although the magnitude of this effect does not compare favourably to other TB vaccine candidates currently under consideration.

The reasons for suboptimal T cell proliferation and IFN-γ production induced by ICM could be multiple. This may be due to the nature of receptors and cells that the ICM encounter following immunisation. In a cell binding assay, ICM were shown to efficiently bind to the surface of a mixed population of dendritic cells (DC) and macrophages *in vitro* and therefore, could be expected to do so *in vivo* as well. Indeed, the Fc receptors are present on a range of cells [53] and ICM could have been internalised by cells other than dendritic cells, with a lesser capacity for T cell priming. Engagement of Fc receptors on dendritic cells leads to cell activation and increased levels of MHC-II and the costimulatory molecules CD80 and CD86, but it is unclear if macrophages are stimulated in a similar manner by the IC. In addition, different Fc receptors are usually present on the surface of both macrophages and dendritic cells, some of which are activating while others may be inhibitory [54], [55]. Evidence from published studies suggests that IC are efficient at inducing CD4^+^ and CD8^+^ T cells and that they enhance antigen processing [9], [52], [56], but that this could be largely dependent on the size of IC and the identity of the Fc receptors they target [57]. If IC are too large, uptake could occur by phagocytic rather than endocytic (i.e. Fc-receptor mediated) mechanisms, and that could determine the efficiency of antigen presentation and the nature of the immune response induced. Only DC but not macrophages can present antigens in the context of MHC I after IC uptake, and considering that CD8^+^ T cell responses may be important for immunity against intracellular bacterial pathogens such as MTB [58], [59], a selective ICM targeting to these cells may be critically important to induce such immune responses.

However, this study does provide conclusive evidence that it is possible to make self-adjuvanting immunogens that can induce strong antibody responses. Given the versatility of the ICM delivery platform, it would be of interest to further test this approach against pathogens which require predominantly humoral rather than cellular immune responses for protection. Indeed, the vast majority of the currently licensed vaccines rely on antibodies as the key component of the protective immune response [60]. In fact, to our knowledge, BCG and Herpes zoster are the only vaccines that rely almost exclusively on cellular rather than humoral immune responses for protection. Thus, the advantage of ICM over other vaccine approaches is that they can induce antibody responses without the use of adjuvants, live vectors or attenuated or inactivated organisms, making their use safe and simplifying the licensing process. The ICM approach is generic and could be easily adapted for various pathogens in two possible ways; 1. Using oligomeric antigens from those pathogens, on their own or fused to additional protective antigens, and a mAb against the oligomeric antigen; 2. using Acr antigen and the anti-Acr mAb as a generic platform, to which antigens from other pathogens could be linked. In both instances, the antibody would have to be fully ‘humanised’ to be applicable in human subjects. On the other hand, Acr, like most heat shock proteins, is a molecular adjuvant in its own right, which is an added advantage of the latter approach. Examples of infections where the ICM could be tested may include HIV, rabies and Influenza, where rapid interception of viruses at the port of entry by pre-existing antibodies appears to be the only realistic way of preventing infection.
